# Association between iron content in grey matter nuclei and functional outcome in patients with acute ischaemic stroke: A quantitative susceptibility mapping study

**DOI:** 10.1111/ene.16531

**Published:** 2024-10-26

**Authors:** Yaqi Chen, Yue Ming, Chen Ye, Shuai Jiang, Jiongxing Wu, Huan Wang, Keying Wu, Shihong Zhang, Bo Wu, Jiayu Sun, Deren Wang

**Affiliations:** ^1^ Department of Neurology, West China Hospital Sichuan University Chengdu China; ^2^ Center of Cerebrovascular Diseases, West China Hospital Sichuan University Chengdu China; ^3^ Department of Radiology, West China Hospital Sichuan University Chengdu China

**Keywords:** grey matter nuclei, iron, ischaemic stroke, prognosis, quantitative susceptibility mapping

## Abstract

**Background and purpose:**

This study aimed to investigate the association between iron content in grey matter (GM) nuclei and functional outcome in acute ischaemic stroke (AIS) patients utilizing quantitative susceptibility mapping.

**Methods:**

Forty AIS patients and 40 age‐, sex‐ and education‐matched healthy controls underwent quantitative susceptibility mapping to assess susceptibility values, which are positively correlated with iron content, in the caudate nucleus, putamen, globus pallidus, thalamus, red nucleus and substantia nigra. The nuclei on the contralateral side were measured in AIS patients to minimize confounding due to oedema or haemorrhage. Functional outcome was determined by the modified Rankin Scale (mRS) score at 3 months after stroke. Poor outcome was defined as mRS >2, whilst a good outcome was defined as ≤2.

**Results:**

Susceptibility values were significantly higher in most contralateral GM nuclei in AIS patients than in the corresponding left or right nuclei in healthy controls. AIS patients with poor outcome showed significantly lower susceptibility value than those with good outcome in the contralateral caudate nucleus, but no significant differences were observed in other GM nuclei. Binary logistic regression analysis revealed a significant association between the susceptibility value of the contralateral caudate nucleus and poor outcome after adjustment for confounders (adjusted odds ratio 0.692, 95% confidence interval 0.486–0.986, *p* = 0.042). Receiver operating characteristic curve analysis showed an acceptable ability of the susceptibility value of the contralateral caudate nucleus to predict poor outcome (area under the curve 0.740, *p* = 0.013).

**Conclusions:**

Lower iron content in the contralateral caudate nucleus was associated with poor functional outcome in AIS patients.

## INTRODUCTION

Ischaemic stroke is one of the most frequent cerebrovascular diseases and a leading cause of abnormal brain function resulting in disability, loss of independence and even death worldwide [1, 2, 3]. Early identification of patients at higher risk for poor recovery or complications is crucial to guide timely and effective interventions [4], yet conventional clinical factors are not sufficiently sensitive or specific to predict stroke prognosis [5, 6]. Recent studies have linked elevated levels of ferritin, an iron‐carrying protein, in serum and cerebrospinal fluid to worse stroke outcome [7, 8, 9]. This connection is consistent with the understanding that ischaemic events cause excessive release of iron into extracellular spaces in the brain, where it can contribute to neuronal damage [10, 11]. However, ferritin is an acute‐phase reactant whose level can rise independently of stroke, and it is only one of the forms in which iron can be present in the brain, making it a less reliable indicator of brain iron levels for the purpose of predicting stroke recovery [12].

Given the established links between excessive extracellular iron in the brain and occurrence of acute ischaemic stroke (AIS) [13, 14], it was considered whether an appropriate indicator of brain iron might predict functional outcome after stroke. Quantitative susceptibility mapping (QSM) is currently regarded as the most effective magnetic resonance imaging (MRI) sequence for accurately estimating tissue susceptibility values, which strongly correlate with iron levels [15, 16]. Whilst other imaging techniques, such as T2*‐weighted imaging (T2*WI), susceptibility‐weighted imaging and R2* relaxometry, can also reflect iron content, each has notable limitations. For instance, T2*WI is a two‐dimensional technique with poor resolution and low sensitivity. Susceptibility‐weighted imaging, though offering high spatial resolution in three dimensions, remains non‐quantitative and is affected by blooming artifacts. R2* relaxometry provides quantitative information on iron levels, but its accuracy is compromised by factors such as geometric orientation and magnetic field inhomogeneities [17, 18]. In contrast, QSM provides a robust and reliable means of quantifying iron as it can reconstruct susceptibility independently of structural geometry and is unaffected by blooming artifacts [16], which makes it the optimal method for accurately assessing brain iron content.

The iron content in grey matter (GM) nuclei was our focus due to the previous finding of its essential role in neurodegenerative diseases, such as Alzheimer's, Parkinson's or Huntington's diseases [19], as well as in stroke patients with long‐term cerebral ischaemia [20, 21]. In particular, increased iron content in the putamen, as measured by QSM, has been associated with greater neurological deficit in stroke patients with long‐term ischaemic symptoms [22]. This suggests that iron dysregulation in GM nuclei might relate to stroke severity and outcome, potentially serving as a prognostic biomarker.

Therefore, in this study, susceptibility values of GM nuclei were compared between AIS patients and appropriately matched healthy controls (HCs) using QSM. Additionally, the association between susceptibility values of GM nuclei and functional outcome was investigated in an effort to identify a prognostic biomarker that reliably reflects brain iron levels. This study aimed to improve the predictive ability for stroke prognosis, enabling more personalized and effective management strategies for stroke patients.

## METHODS

The study was approved by the Ethics Committee on Biomedical Research, West China Hospital of Sichuan University (2019 323), conducted in accordance with the Declaration of Helsinki and reported according to the Strengthening the Reporting of Observational Studies in Epidemiology (STROBE) guidelines [23]. All participants or their legal guardians provided written informed consent.

### Subjects

A consecutive series of patients who were admitted to the Neurology Department of West China Hospital between February 2022 and December 2023 were prospectively enrolled in this study if they were at least 18 years old and diagnosed with AIS based on clinical manifestations, which was confirmed by MRI within 2 weeks after stroke onset. Patients were excluded if they had any of the following: (i) severe cerebral oedema or haemorrhagic transformation, which was classified as parenchymal haematoma according to ECASS III criteria [24]; (ii) bilateral ischaemic stroke; (iii) history of stroke, Parkinson's disease, dementia, multiple sclerosis, brain injury, brain tumour, brain calcification or other neurological disease; (iv) history of depression, anxiety, schizophrenia, moderate or severe secondary anaemia, autoimmune disease, malignant tumour or other severe systemic disease; (v) history of therapy involving iron supplementation or sedatives; or (vi) magnetic resonance images with severe artifacts. Patients were also excluded if they did not complete follow‐up at 3 months after stroke onset.

Healthy controls at least 18 years old were recruited from the local community. Enrolled HCs had no neurological or psychiatric disorders, no severe systemic diseases and no obvious cerebral lesions on MRI.

### Clinicodemographic characteristics of patients

Patient demographics and clinical features at baseline were recorded including age, sex, education level, history of hypertension, history of diabetes mellitus, current smoking status, alcohol consumption, stroke severity measured by the National Institutes of Health Stroke Scale (NIHSS), systolic and diastolic blood pressure from the initial evaluation, time from onset until MRI scan and duration of hospitalization. Data on reperfusion treatments during hospitalization, including intravenous thrombolysis and/or endovascular therapy, were collected. Infarct location was determined from MRI, and infarct volume was estimated from diffusion‐weighted imaging using the Fiji package in ImageJ 1.54f (National Institutes of Health, Bethesda, MD, USA). Stroke aetiology was classified according to the TOAST criteria [25]. Early cerebral ischaemic change was assessed in terms of the Alberta Stroke Programme Early Computed Tomography Score [26] from computed tomography without enhancement. Collateral circulation was assessed using the Tan score [27] from computed tomography angiography: a Tan score of 0–1 was defined as poor collateral circulation.

### Outcome of patients

At 3 months after stroke onset, patients or family members were followed up through telephone interview using the modified Rankin Scale (mRS) score. The functional outcome classified a poor outcome as mRS >2 and a good one as mRS ≤2 [28].

### Magnetic resonance imaging protocol

All enrolled subjects underwent MRI experiments on a 3.0‐T MR scanner (SIGNA™ Premier, GE Medical Systems) equipped with a 32‐channel phase‐array head coil. Regular MRI sequences including T1‐weighted imaging, T2‐weighted imaging, T2 fluid‐attenuated inversion recovery and diffusion‐weighted imaging as well as high‐resolution 3D T1‐weighted structural images and 3D spoiled gradient echo‐based QSM images were scanned for each participant. 3D T1‐weighted images (hereafter referred to as ‘T1 structural images’) were obtained using a brain volume sequence with the following parameters: repetition time (TR) 7.2 ms; echo time (TE) 3.0 ms; field of view (FOV) 256 mm × 256 mm; matrix size 256 mm × 256 mm; slice thickness 1.0 mm; number of slices 152; flip angle (FA) 12°; and scanning time 223 s. The 3D spoiled gradient echo‐based QSM images were obtained with the following parameters: TR 39.9 ms; first TE 3.0 ms; TE interval 3.1 ms; number of TEs 12; FOV 240 mm × 240 mm; matrix size 240 mm × 216 mm; number of slices 3072; FA 20°; and scanning time 400 s.

### Imaging analysis

Quantitative susceptibility mapping images were reconstructed using STI Suite 3.0 (http://people.eecs.berkeley.edu/~chunlei.liu/software.html) embedded in MATLAB (MathWorks, Natick, MA, USA). Laplacian unwrapping algorithms were used to generate unwrapped phase images [29], and brain tissue was extracted in order to create a brain mask. Background fields were removed using the ‘variable‐kernel sophisticated harmonic artifact reduction for phase data’ (V‐SHARP) method with a maximum kernel of 12 mm [30]. Final QSM images were obtained through the iterative least‐squares (iLSQR) algorithm [31].

**FIGURE 1:**
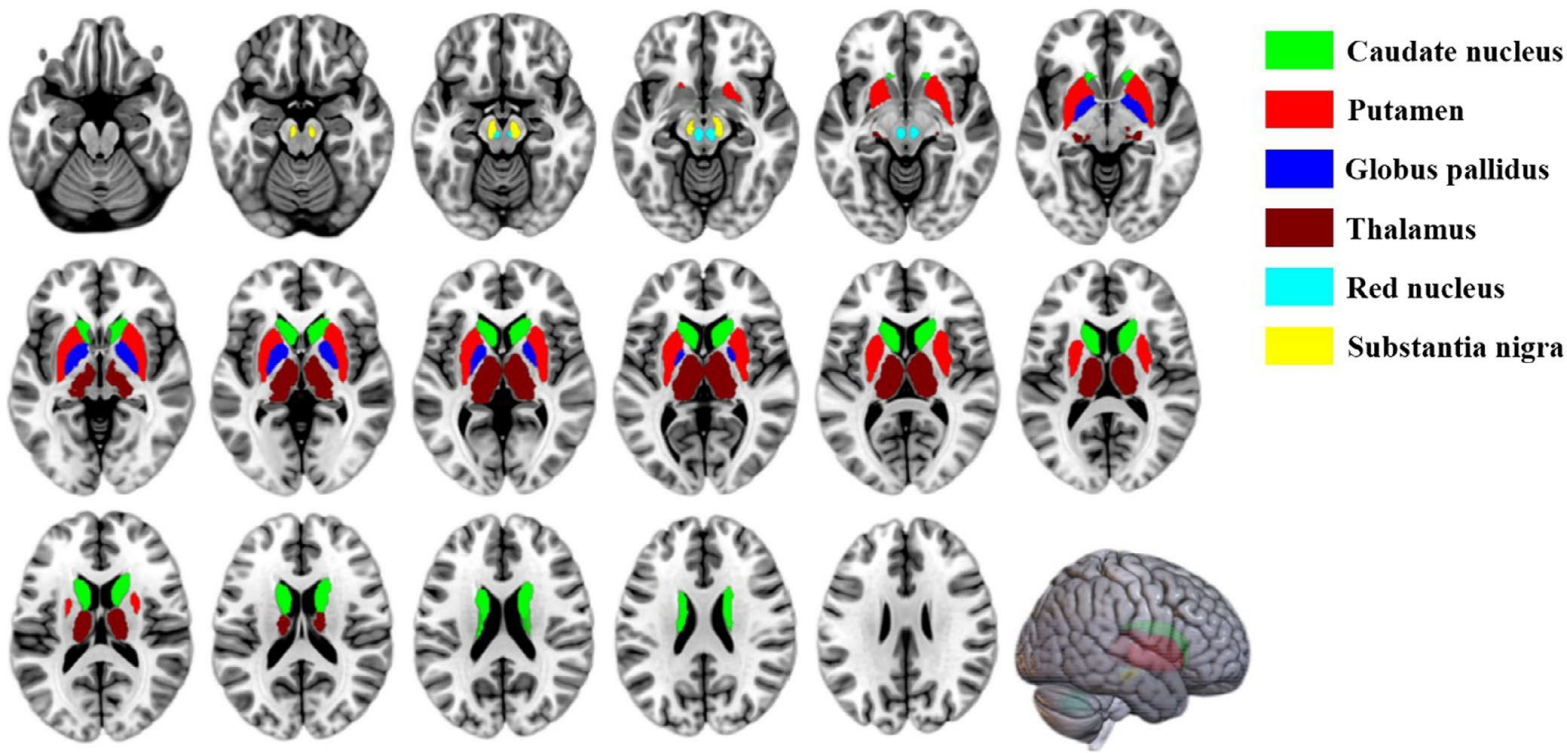
Regions of interest in grey matter nuclei depicted on T1 structural images.

Regions of interest (ROIs) were defined covering the following GM nuclei: caudate nucleus, putamen, globus pallidus, thalamus, red nucleus and substantia nigra. ROIs were drawn on the left and right sides of HCs but only on the contralateral side of patients in order to minimize confounding due to oedema or haemorrhage. The nuclei were identified on T1 structural images using the Automated Anatomical Labelling Atlas 3 (https://www.gin.cnrs.fr/en/tools/aal/) (Figure 1). Susceptibility values, which vary in direct proportion to iron content [32], were extracted for each ROI based on the co‐registration of QSM images and T1 structural images that had been normalized to standard Montreal Neurological Institute 152 space using Advanced Normalization Tools (https://github.com/ANTsX/ANTs). Frontal white matter (fWM) has low iron content and therefore can help correct for inter‐individual variation in ROIs [33]. The volume of interest of fWM spanning five adjacent T1 structural image slices was drawn by an experienced neurologist using MRIcron (https://www.nitrc.org/projects/mricron/) and then warped to QSM. The susceptibility value of the corresponding fWM was subtracted from the susceptibility values of ROIs in each participant.

### Statistical analysis

Continuous data were reported as mean ± standard deviation if normally distributed or as median (interquartile range) if skewed. Categorical data were expressed as *n* (%). For clinicodemographic variables, intergroup differences were assessed for significance using Student's *t* test or the Mann–Whitney *U* test in the case of continuous data or using the chi‐squared test or Fisher's exact test in the case of categorical data. For susceptibility values, intergroup differences were assessed using one‐way analysis of variance, the independent‐samples *t* test or the rank‐sum test.

Binary logistic regression analyses were performed to examine the associations between susceptibility values in contralateral GM nuclei and poor outcomes, with odds ratios (OR) and 95% confidence intervals (CI) calculated. The model was adjusted for demographic variables, including age and sex, which are recognized prognostic factors, as well as other clinical confounders that had a *p* value of less than 0.10 in the bivariate analysis of baseline characteristics. The ability of susceptibility value in contralateral GM nuclei, either on its own or combined with conventional clinical factors (age, sex and variables that had a *p* value of less than 0.10 in the bivariate analysis), to predict poor outcome was assessed using receiver operating characteristic curve analyses [34].

Statistical analyses were carried out in SPSS 24.0 (IBM, Armonk, NY, USA) and MedCalc Statistical Software 22.014 (MedCalc Software, Ostend, Belgium). Results were considered statistically significant if associated with a two‐tailed *p* < 0.05. Figures were prepared using GraphPad Prism 8.0 (GraphPad Software, San Diego, CA, USA).

## RESULTS

### Baseline characteristics

The final analysis included 40 AIS patients and 40 HCs with similar distribution in age, sex and education level (Table S1). Of the 40 patients, nine (22.5%) showed poor outcome at 3 months after stroke onset (Table 1). Compared to patients with good outcome, those with poor outcome had significantly higher NIHSS at admission, remained significantly longer in hospital, and were more likely to have experienced stroke caused by large‐artery atherosclerosis.

**TABLE 1:** Clinicodemographic characteristics of acute ischaemic stroke patients stratified by outcome.

Characteristic	All (*N* = 40)	Good outcome (*n* = 31)	Poor outcome (*n* = 9)	*p*
Age, years	67.5 (53.0–71.0)	69.0 (53.0–72.0)	64.0 (51.0–68.5)	0.236
Women	14 (35)	12 (38.7)	2 (22.2)	0.453
Years of education	9 (6–12)	9 (6–12)	6 (6–14)	0.932
Hypertension	22 (55)	17 (54.8)	5 (55.6)	1.000
Diabetes mellitus	7 (17.5)	5 (16.1)	2 (22.2)	0.645
Current smoking	13 (32.5)	9 (29.0)	4 (44.4)	0.437
Alcohol consumption	9 (22.5)	5 (16.1)	4 (44.4)	0.168
Systolic blood pressure, mmHg	152.13 ± 21.24	151.13 ± 21.60	155.56 ± 20.80	0.589
Diastolic blood pressure, mmHg	88.58 ± 15.56	87.65 ± 16.21	91.78 ± 13.45	0.490
NIHSS score	4 (2.25–10)	3 (2–6)	10 (3.5–13.5)	**0.019**
Infarct location				0.717
Anterior circulation	24 (60)	18 (58.1)	6 (66.7)	
Posterior circulation	16 (40)	13 (41.9)	3 (33.3)	
Reperfusion therapy	21 (52.5)	15 (48.4)	6 (66.6)	0.457
Stroke aetiology				**0.003**
Large‐artery atherosclerosis	16 (40)	8 (25.8)	8 (88.9)	
Cardioembolism	9 (22.5)	8 (25.8)	1 (11.1)	
Small‐artery occlusion	15 (37.5)	15 (48.4)	0 (0)	
ASPECTS	8 (7–10)	8 (7–10)	7 (6.5–10)	0.483
Poor collateral circulation	17 (42.5)	12 (38.7)	5 (55.6)	0.456
Infarct volume, mL	2.89 (0.70–7.43)	1.73 (0.54–7.01)	4.86 (3.02–23.86)	0.225
Haemorrhagic transformation	5 (12.5)	3 (9.7)	2 (22.2)	0.311
Time from onset until QSM, h	171.88 ± 79.77	175.35 ± 81.49	159.89 ± 76.89	0.615
Duration of hospitalization, days	8 (7–12)	7 (6–10)	11 (9–12.5)	**0.033**

*Note*: Values are *n* (%), median (interquartile range) or mean ± SD, unless otherwise noted. Bold values denote statistical significance at the *p* < 0.05.

Abbreviations: ASPECTS, Alberta Stroke Programme Early Computed Tomography Score; NIHSS, National Institutes of Health Stroke Scale; QSM, quantitative susceptibility mapping.

### Comparisons of susceptibility values of GM nuclei between AIS patients and HCs

**FIGURE 2:**
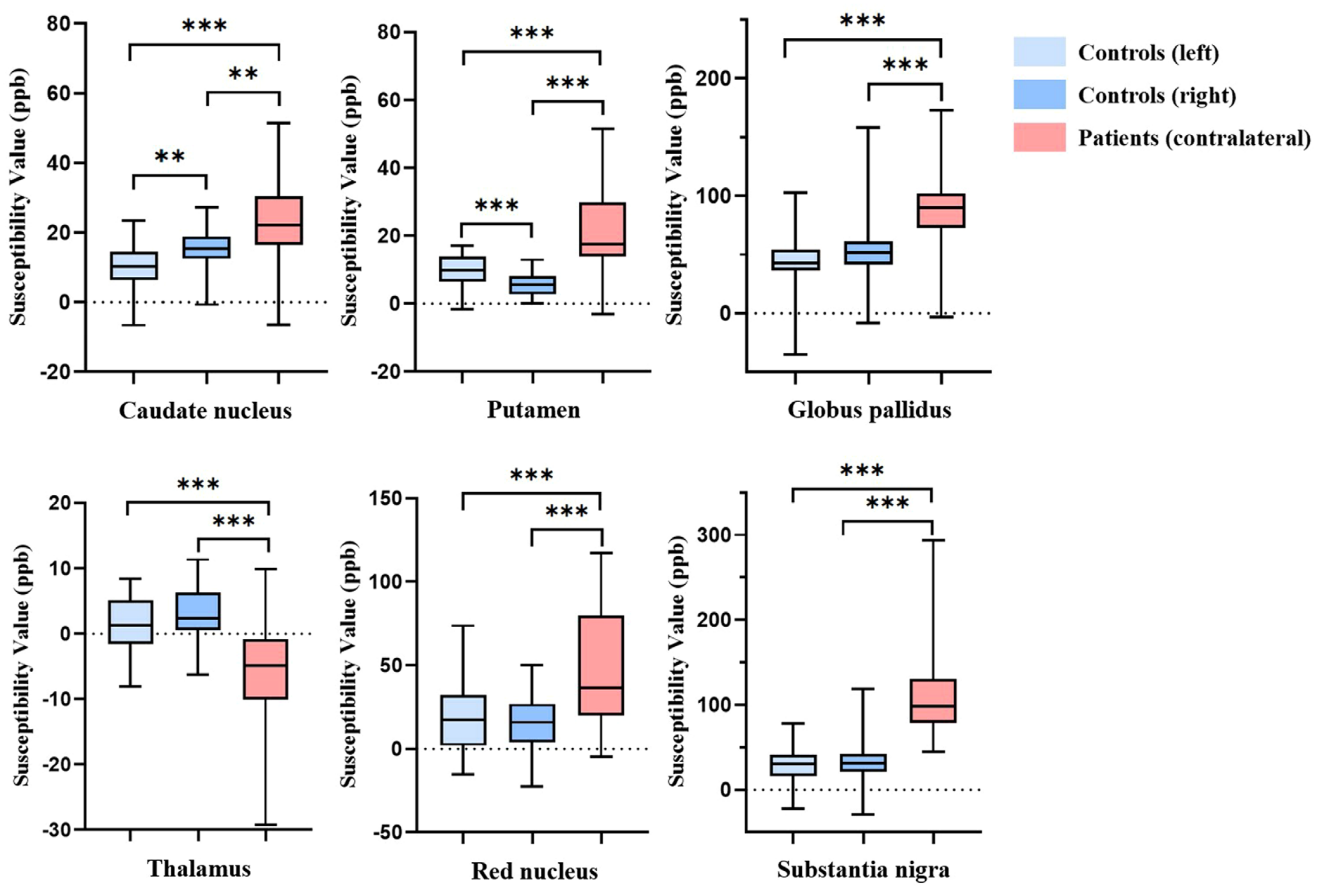
Comparison of susceptibility values of grey matter nuclei between healthy controls and acute ischaemic stroke patients. ***p < 0.001, **p < 0.01.

Susceptibility values were significantly higher on the contralateral side of AIS patients than on the left and right sides of HCs in all GM nuclei except the thalamus, where patients showed lower susceptibility value (Figure 2, Table S2). In HCs, susceptibility values differed significantly between the left and right side of the caudate nucleus and putamen.

### Susceptibility values of contralateral GM nuclei and poor outcome

**FIGURE 3:**
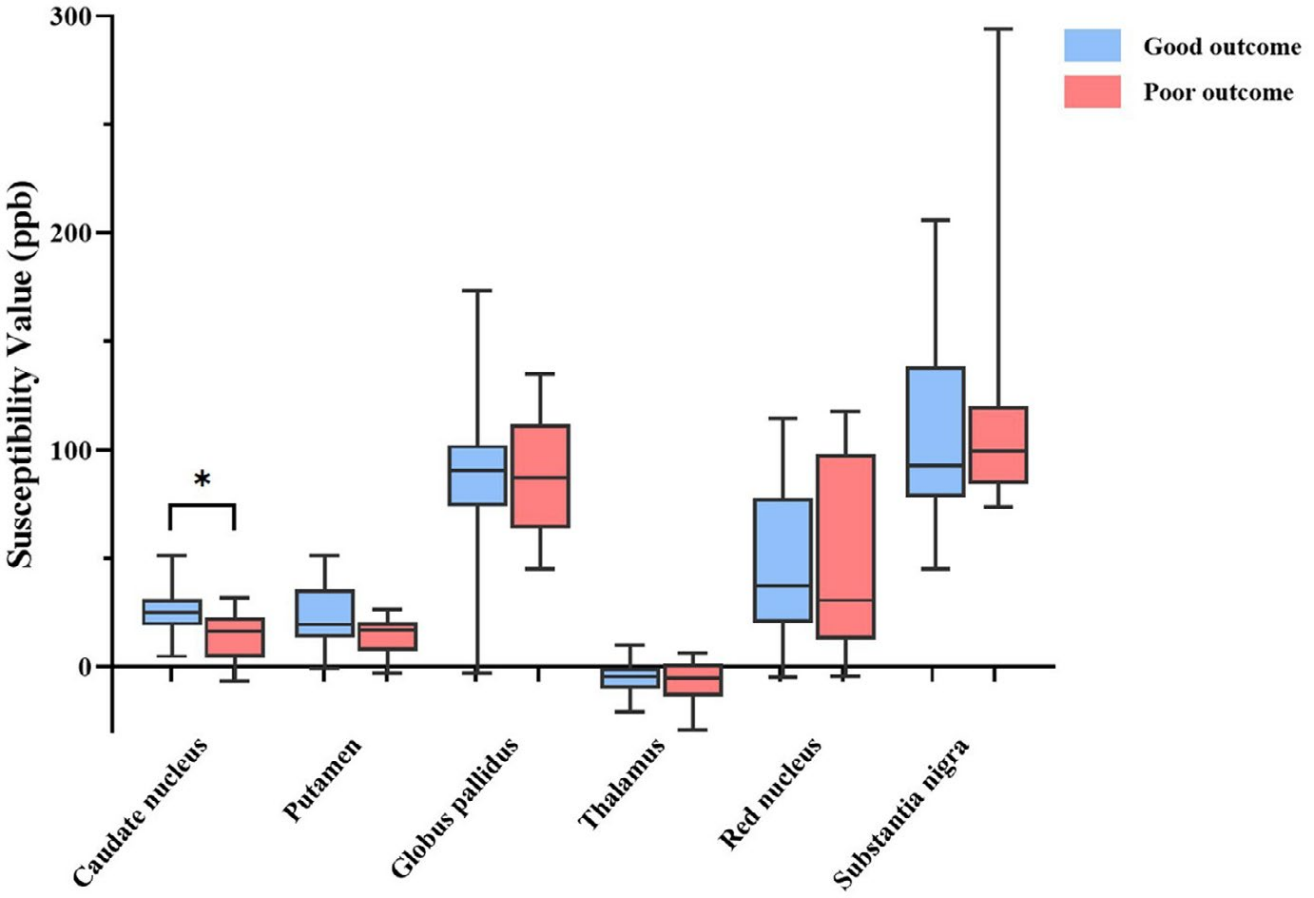
Comparison of susceptibility values of contralateral grey matter nuclei between acute ischaemic stroke patients with good or poor outcome. *p < 0.05.

Patients with poor outcome exhibited significantly lower susceptibility value of the contralateral caudate nucleus compared to those with good outcome, whilst similar susceptibility values were found in other GM nuclei between groups (Table 2, Figure 3). Of all contralateral GM nuclei in patients, only the susceptibility value of the caudate nucleus was independently associated with poor outcome after controlling for age, sex, NIHSS, stroke aetiology and duration of hospitalization (adjusted OR 0.692, 95% CI 0.486–0.986, *p* = 0.042; Table 3).

**TABLE 2:** Comparison of susceptibility values (in ppb) of contralateral grey matter nuclei between acute ischaemic stroke patients with good or poor outcome.

Nucleus	Good outcome (*n* = 31)	Poor outcome (*n* = 9)	*p*
Caudate nucleus	25.03 ± 10.62	14.31 ± 12.01	**0.013**
Putamen	23.42 ± 14.10	14.37 ± 9.71	0.080
Globus pallidus	90.60 (74.00–102.39)	87.25 (63.85–111.55)	0.545
Thalamus	−4.86 ± 7.80	−7.12 ± 10.92	0.489
Red nucleus	46.32 ± 33.94	49.06 ± 45.43	0.845
Substantia nigra	92.95 (78.31–138.55)	99.50 (84.41–119.97)	0.545

*Note*: Values are median (interquartile range) or mean ± SD, unless otherwise noted. Bold values denote statistical significance at the *p* < 0.05.

**TABLE 3:** Associations between susceptibility values of contralateral grey matter nuclei and poor outcome in acute ischaemic stroke patients.

Nucleus	OR (95% CI)^a^	*p*
Caudate nucleus	0.692 (0.486–0.986)	**0.042**
Putamen	0.955 (0.841–1.084)	0.477
Globus pallidus	1.063 (0.990–1.140)	0.091
Thalamus	0.910 (0.759–1.091)	0.310
Red nucleus	0.994 (0.970–1.020)	0.661
Substantia nigra	1.024 (0.981–1.070)	0.275

Abbreviations: CI, confidence interval; NIHSS, National Institutes of Health Stroke Scale; OR, odds ratio. Bold values denote statistical significance at the *p* < 0.05.

^a^ The regression model adjusted for age, sex, NIHSS score, stroke aetiology and duration of hospitalization.

The susceptibility value of the contralateral caudate nucleus showed an acceptable ability to predict poor outcome with an area under the curve (AUC) of 0.740 (95% CI 0.577–0.866, *p* = 0.013; Table 4). However, the inclusion of this value provided a non‐significant improvement in the predictive efficiency of a model involving conventional clinical factors (AUC 0.964 vs. 0.918, *p* = 0.349).

**TABLE 4:** Ability of the susceptibility value of the contralateral caudate nucleus to predict poor outcome in acute ischaemic stroke patients.

Predictor	AUC (95% CI)	*p*	Sensitivity	Specificity	PPV	NPV
Susceptibility value of contralateral caudate nucleus	0.740 (0.577–0.866)	**0.013**	88.89	51.61	44.05	91.55
Conventional clinical factors^a^	0.918 (0.786–0.981)	**<0.001**	66.67	100	100	87.50
Susceptibility value + conventional clinical factors	0.964 (0.852–0.998)	**<0.001**	100	83.87	76.95	100

Abbreviations: AUC, area under receiver operating characteristic curve; CI, confidence interval; NIHSS, National Institutes of Health Stroke Scale; NPV, negative predictive value; PPV, positive predictive value. Bold values denote statistical significance at the *p* < 0.05.

^a^ Age, sex, NIHSS score, stroke aetiology and duration of hospitalization.

## DISCUSSION

In this study, QSM‐derived susceptibility values were used to investigate the association between iron content in GM nuclei and functional outcome in AIS patients. The results demonstrated higher susceptibility values of most GM nuclei on the contralateral side in AIS patients than those on the left or right side in HCs. In addition, a lower susceptibility value of the contralateral caudate nucleus was independently associated with poor functional outcome, and might serve as a potential marker to predict poor stroke prognosis. To our knowledge, this is the first study that utilizes QSM to determine whether iron content in GM nuclei during the acute phase of ischaemic stroke might be a useful predictor of prognosis.

Excessive iron accumulation was found in contralateral GM nucleus subregions far from the infarct lesion in AIS patients, suggesting a broad iron dysregulation after stroke. This is in line with a previous basic study that demonstrated the increase of iron levels in affected rat brain tissues after permanent focal ischaemia [10]. When stroke occurs, vascular endothelial cells become ischaemic and hypoxic, leading to disruption of the blood–brain barrier (BBB) [35, 36]. In parallel, ischaemia‐induced acidosis as well as formation of reactive oxygen species and nitric oxide induce the release of free iron from ferritin [10]. These processes release excessive iron into extracellular spaces, where it can harm neurons [37, 38, 39]. However, whether contralateral iron deposition causes neuronal excitotoxicity should be explored in the future, since direct evidence of such a connection has yet to be reported [13]. In contrast to our finding, a clinical controlled study found higher iron content only in the contralateral putamen but not in other GM nuclei of patients with long‐term ischaemic stroke symptoms caused by unilateral middle cerebral artery stenosis or occlusion [21]. This discrepancy might reflect differences in the types of enrolled stroke patients, and it might be attributed to the different scanning timepoints of patient evaluation: our study examined patients within 2 weeks of stroke onset, whereas the previous study examined patients at least 1 month after stroke onset. Longitudinal studies tracking brain iron levels at different stroke stages are necessary to clarify this discrepancy.

Our results implied that lower iron content in the contralateral caudate nucleus far from ischaemic lesions was related to poor functional outcome, which was consistent with a mouse model study of ischaemic stroke linking lower iron content in the lesion side of the brain to worse long‐term recovery [40]. They observed that iron deficiency could promote glial hyperplasia and inhibit the migration and differentiation of neural stem cells, delaying neuronal regeneration after ischaemic injury. However, the exact mechanism of the relevance between iron content in the contralateral caudate nucleus and stroke outcomes remains unknown due to limited evidence, with only preliminary clues currently available. Human research utilizing positron emission tomography or computed tomographic perfusion has identified cerebral blood flow reduction and BBB damage in the hemisphere contralateral to the infarct lesion [41, 42], possibly due to transhemispheric diaschisis [43]. Also, studies in rats have shown that cerebral blood flow reductions are more pronounced in forebrain regions, including the caudate nucleus, compared to diencephalic areas like the thalamus and substantia nigra during global ischaemia [44]. Thus, it is speculated that the contralateral caudate nucleus is particularly vulnerable to iron dysregulation resulting from hypoxia‐ischaemia insult and BBB disruption, especially in the context of reduced blood flow following unilateral ischaemic stroke. This dysregulation may contribute to local neuronal damage, ultimately leading to poorer prognosis. Future studies incorporating clinical imaging and pathophysiological evidence are needed to validate this hypothesis. Future research could explore oxygen metabolism in non‐infarcted GM nuclei following focal ischaemia, using oxygen extraction fraction as an indicator of brain tissue viability and function [45, 46]. Investigating its relationship with localized iron content may help elucidate the underlying mechanism of our finding. Moreover, our finding aligns with previous research showing that iron concentration within the infarct lesion correlates with neurological outcomes [47]. Therefore, conducting a whole‐brain analysis would be valuable to determine the relationship between iron levels in other brain regions and stroke prognosis, and to assess whether interactions exist between iron content in different brain regions and their impact on outcomes.

Our finding that lower iron content in the contralateral caudate nucleus was associated with poor outcome seems to contradict previous observations that higher levels of the iron carrier ferritin in the blood were associated with worse prognosis [7, 9]. These two observations are not necessarily in conflict with each other, however. One reason is that whilst ferritin is known to release free iron into the brain parenchyma after stroke‐induced injury of the BBB [12], another iron carrier, transferrin, can reduce iron availability and mitigate ischaemic injury [48]. Another reason is that the excess iron causing brain injury may not originate in the blood [13]. These considerations highlight the need to elucidate the potentially complex competing processes that regulate iron levels in the brain after AIS. They also highlight the importance of relying on actual iron concentration in the brain as reflected in susceptibility values or other reliable imaging markers, instead of surrogate markers such as ferritin level in blood or cerebrospinal fluid.

Our findings should be interpreted with caution due to several limitations. First, patients with severe neurological deficits or unstable consciousness were excluded, as they were unable to tolerate prolonged MRI scans. This may have introduced selection bias. Second, the extended scan duration increases the likelihood of even minor head movements, which can reduce MRI and QSM image quality, potentially compromising the accuracy of the susceptibility measurements. Additionally, susceptibility values derived from QSM are influenced not only by iron but also by other paramagnetic substances, such as manganese, copper, haemosiderin and deoxygenated haemoglobin, which could contribute to measurement bias [49]. Finally, given the potential biases mentioned and the limitations of our single‐centre study with a relatively small sample size, further studies are necessary to confirm our findings.

## CONCLUSION

Our prospective study demonstrates that AIS involves a significant increase in iron content in most contralateral GM nuclei, and that lower iron content in the contralateral caudate nucleus is independently associated with poor functional outcome. These findings highlight the utility of QSM in elucidating ischaemic injury pathways and identifying prognostic markers that directly reflect brain iron content. Further studies with larger samples and longer follow‐up are warranted to validate our findings and explore the relationship between the dynamic variation of iron content in GM nuclei and stroke outcome.

## AUTHOR CONTRIBUTIONS

**Yaqi Chen:** Conceptualization; methodology; software; investigation; formal analysis; writing – original draft. **Yue Ming:** Methodology; software; data curation; visualization; formal analysis. **Chen Ye:** Data curation; investigation; software. **Shuai Jiang:** Software; investigation; data curation. **Jiongxing Wu:** Visualization; formal analysis; methodology. **Huan Wang:** Data curation; investigation; visualization. **Keying Wu:** Data curation; investigation; visualization. **Shihong Zhang:** Data curation; investigation. **Bo Wu:** Data curation; investigation. **Jiayu Sun:** Conceptualization; methodology; software; data curation; supervision. **Deren Wang:** Conceptualization; methodology; software; funding acquisition; project administration; writing – review and editing.

## FUNDING INFORMATION

DW was supported by the National Natural Science Foundation of China (grant no. 81870923 and 82271331) and the 1·3·5 Project for Disciplines of Excellence‐Clinical Research Incubation Project of West China Hospital (grant no. 2018HXFH041).

## CONFLICT OF INTEREST STATEMENT

All authors declare no potential conflicts of interest.

## Supporting information


Table S1–S2.

## Data Availability

The data that support the findings of this study are available from the corresponding author upon reasonable request.
