# Supplementary material for: Association between iron content in grey matter nuclei and functional outcome in patients with acute ischaemic stroke: A quantitative susceptibility mapping study
Source: Eur J Neurol. 2024 Oct 26;32(1):e16531. doi: 10.1111/ene.16531 (PMC11622281; doi:10.1111/ene.16531)
Supplement: Supplementary file 1 — Table S1–S2. [file ENE-32-e16531-s001.docx]

**Supplementary Table S1. Comparison of Baseline Characteristics between Healthy Controls and Acute Ischemic Stroke Patients.**

| Characteristic | Healthy Controls  (n = 40) | Patients  (n = 40) | *P* |
| --- | --- | --- | --- |
| Age, y | 59.98 ± 7.45 | 63.30 ± 10.80 | 0.113 |
| Women | 15 (37.5) | 14 (35) | 0.816 |
| Years of education | 9 (6.25-15) | 9 (6-12) | 0.195 |

Values are mean ± SD, n (%) or median (interquartile range), unless otherwise noted.

**Supplementary Table S2. Comparison of Susceptibility Values (in ppb) of Gray Matter Nuclei between Healthy Controls and Acute Ischemic Stroke Patients.**

| **Nucleus** | **Healthy controls** | | **Patients** | ***P* (post hoc)** | | |
| --- | --- | --- | --- | --- | --- | --- |
|  | Left side | Right side | Contralateral | Left *vs* Right in controls | Controls (left) *vs* Patients | Controls (right) *vs* Patients |
| Caudate nucleus | 10.08 ± 6.46 | 15.52 ± 6.10 | 22.62 ± 11.70 | 0.001 ^b^ | <0.001 ^b^ | 0.004 ^b^ |
| Putamen | 9.75 ± 4.51 | 5.58 ± 3.11 | 21.38 ± 13.67 | <0.001 ^b^ | <0.001 ^b^ | <0.001 ^b^ |
| Globus pallidus | 42.73 ± 21.93 | 51.48 ± 24.72 | 90.93 ± 34.29 | 0.267 ^a^ | <0.001 ^a^ | <0.001 ^a^ |
| Thalamus | 1.34 ± 4.25 | 3.07 ± 4.34 | -5.36 ± 8.50 | 0.213 ^b^ | <0.001 ^b^ | <0.001 ^b^ |
| Red nucleus | 19.46 ± 18.52 | 15.89 ± 17.50 | 46.94 ± 36.20 | 0.759 ^b^ | <0.001 ^b^ | <0.001 ^b^ |
| Substantia nigra | 30.52 ± 19.96 | 33.87 ± 25.52 | 109.32 ± 47.57 | 0.886 ^b^ | <0.001 ^b^ | <0.001 ^b^ |

Values are mean ± SD, unless otherwise noted.

^a^ After Bonferroni correction

^b^ After Tamhane’s T2 correction.
